# Multiring basin formation constrains Europa’s ice shell thickness

**DOI:** 10.1126/sciadv.adj8455

**Published:** 2024-03-20

**Authors:** Shigeru Wakita, Brandon C. Johnson, Elizabeth A. Silber, Kelsi N. Singer

**Affiliations:** ^1^ Department of Earth, Atmospheric, and Planetary Sciences, Massachusetts Institute of Technology, Cambridge, MA, USA.; ^2^ Department of Earth, Atmospheric, and Planetary Sciences, Purdue University, West Lafayette, IN, USA.; ^3^ Department of Physics and Astronomy, Purdue University, West Lafayette, IN, USA.; ^4^ Department of Earth Sciences, Western University, London, ON, Canada.; ^5^ Institute for Earth and Space Exploration, Western University, London, ON, Canada.; ^6^ Southwest Research Institute, Boulder, CO, USA.

## Abstract

Jupiter’s moon Europa hosts a subsurface ocean under an ice shell of uncertain thickness. Europa has two multiring basins that exhibit several concentric rings. The formation of these multiring basins is thought to be sensitive to the thickness and thermal structure of the ice shell. Here, we simulate multiring basin forming impacts on Europa finding that a total ice shell greater than 20 kilometers thick is required to reproduce observed ring structures. Thin ice shells (<15 kilometers thick) result in compressional tectonics inconsistent with observed ring structures. Our simulations are also sensitive to the thermal structure of the ice shell and indicate that Europa’s at least 20-kilometer ice shell is composed of a 6- to 8-kilometer-thick conductive lid overlying warm convecting ice. The constraints on Europa’s ice shell structure resulting from this work are directly relevant to our understanding of the potential habitability of Europa.

## Teaser

A thick conductive-convective ice shell existed over the ocean at the formation time of Europa’s multiring basins.

## INTRODUCTION

There is strong evidence that Europa hosts a subsurface ocean (*1*, *2*). Estimates of Europa’s ice shell thickness range from only a few kilometers thick (*3*–*5*) up to a few tens of kilometers thick (*6*, *7*). The thickness of Europa’s ice shell constrains formation mechanisms for various surface features and determines the processes that take place in the ice shell (*8*). The ice shell thickness also determines the degree of tidal heating within the ice shell (*9*) and how exchange may occur between the surface and ocean (*4*, *10*, *11*), both of which are important to understanding the potential habitability of Europa (*12*).

Craters on Europa have long been seen as an important way to constrain ice shell structure. An impact first excavates a transient crater that will collapse under the force of gravity producing the post-impact crater morphology. Note that the morphology we observe today may have experienced relaxation and modification by other post-impact processes. The presence of central peaks within some Europan craters suggest that impacts did not melt through the entire ice shell, implying that the ice shell is at least 3 to 4 km thick (*13*). Crater depth diameter trends on Europa show that craters larger than about 8 km in diameter become shallower than their smaller counterparts (*14*). Simulations of crater formation support the interpretation that the collapse of the transient crater is affected by a weak layer at depth (*14*) and suggest that the conductive portion of the ice shell is ~7 km thick (*15*–*17*). However, these simulations could not distinguish between a weak layer composed of warm convecting ice or liquid water below the upper conductive ice lid (*15*–*17*). Assuming that the transition to multiring structures marks a similar rheologic transition at depth and that this rheologic transition corresponds to the ice-ocean interface, the total ice shell of Europa would be 19 to 25 km thick (*14*).

Callanish and Tyre are Valhalla-type multiring basins that exhibit several concentric ring graben extending to almost 100 km from their centers (*18*–*22*). These multiring basins consist of three regions, a central basin region within the crater rim and inner and outer ring regions beyond the rim (*18*–*20*). Graben, trough-shaped features, are produced by a pair of antithetical normal faults (*23*) and make up the rings in the outer ring region. Basin rings form when the collapse of the transient crater is accommodated by the flow of weak material that underlies a strong lithosphere (*24*–*27*). The inward flow pulls the lithosphere along with it, causing extension and the formation of normal faults. If the lithosphere is sufficiently thin, several concentric graben are expected to form (*25*). Thus, multiring basin formation is sensitive to the target thermomechanical structure (*28*), and simulations of multiring basin formation on Europa can provide insight into its ice shell thickness and thermal structure. We simulated the formation of multiring basins on Europa using the iSALE-2D shock physics code (*29*–*31*). We considered 1.0- to 1.8-km-radius impactors striking a Europa-like target at 15 km/s with a mesh spatial resolution of 50 m. We tested ice shell thicknesses from 10 to 50 km with lithospheric thermal gradients of 34.60 to 17.31 K/km, corresponding to conductive lid of 5 to 10 km. We used the radial strain based on observed graben (*21*, *22*) as the primary constraint on our simulations. Radial strain represents the magnitude of extension accommodated by graben and is calculated by accumulating the radial displacement of the graben from the outside of the basin toward its center (*21*, *22*). Our model setup and material parameters are consistent with previous work (*15*, *17*). Motivated by simulations of lunar multiring basin formation (*26*), we also included a damage model with an exponential dependence on strain (*32*) and a dilatancy model (*33*) that can enhance strain localization and lead to fault-like behavior. More details can be found in Materials and Methods.

## RESULTS

### Multiring basin formed on a thick ice shell

**Fig. 1.**
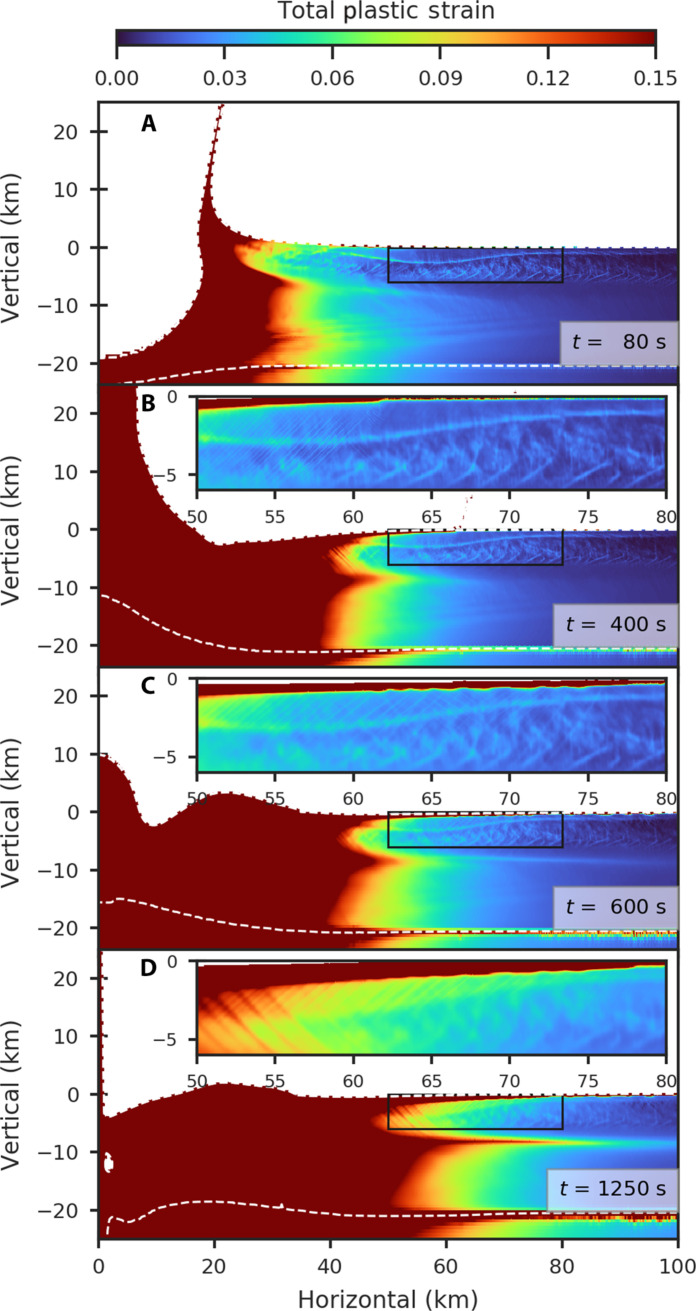
Time series of our fiducial model of the formation of an icy multiring basin. Simulation is of a 1.5-km-radius impactor striking a 20-km-thick ice shell with a 6-km-thick conductive lid. Material is colored according to total plastic strain. White dashed curves indicate the material boundaries (i.e., ice shell and ocean). The insets are a magnified view of the upper 6 km of the conductive ice lid and outer portions of the basin where graben would form. Figure S1 shows the total plastic strain in log scale, and fig. S4 outlines the location of graben at 600 s. Movie S1 is an animation of this figure. (**A**) shows the formation of the transient crater, (**B**) shows the formation of the central uplift, (**C**) shows the formation of faults, and (**D**) represents the enhancement of faults during a secondary phase of collapse (see the main text for details).

Our fiducial model is a 1.5-km-radius impactor striking a target with a 6-km-thick conductive lid and a total ice shell thickness of 20 km at 15 km/s. Note that the 6-km conductive lid corresponds to a heat flow of 86 mW/m^2^ assuming a constant thermal conductivity of 3 W m^−1^ K^−1^ (*15*). While the estimated average impact velocity on Europa is 26 km/s (*34*), crater scaling laws imply that at 26 km/s, an impactor that is 0.7 times smaller than an impactor striking at 15 km/s would produce the same size transient crater and result in formation of a similar basin morphology (*35*). We first focus on the dynamics of crater collapse and the total plastic strain that occurs. Faults in iSALE manifest as regions of localized total plastic strain rather than discrete slip planes. The impact produces a shock wave followed soon after by a rarefaction or release wave. The passage of these waves sets the material in motion excavating a 19-km-deep transient crater (Fig. 1A and fig. S1A). The transient crater collapses under the force of gravity, producing a large central uplift (Fig. 1B and fig. S1B). As the central uplift collapses, the material further from the basin center is still moving inward and small-scale normal faults cutting both toward and away from the basin are forming (Fig. 1C and fig. S1C). The collapse of the central uplift creates another cavity, which, in turn, collapses, resulting in more inward flow. Most of the inward flow of ice is accommodated by weak material at ~8 km depth from the surface, and most of the inward flow of material greater than 50 km from the basin center occurs during the second phase of inward collapse (Fig. 1D and fig. S1D). The second phase of collapse causes the strain on normal faults, which were produced during the first phase of collapse, to increase by a factor of 4 to 5 (Fig. 1, C and D, and fig. S1, C and D). After 2000 s, the crater has settled to its final state (fig. S2A). Compared to the faults that make up the rings observed around Tyre and Callanish (*21*, *22*) (fig. S3), our simulations produce more normal faults with smaller offsets (see fig. S4). Testing at lower resolution suggests that considering higher resolution would enhance strain localization. Considering rheologies that enhance strain localization (*36*, *37*) would also likely result in strain being localized on fewer faults with larger offsets. However, the style of faulting and total strain accommodated by these faults is consistent with observations.

**Fig. 2.**
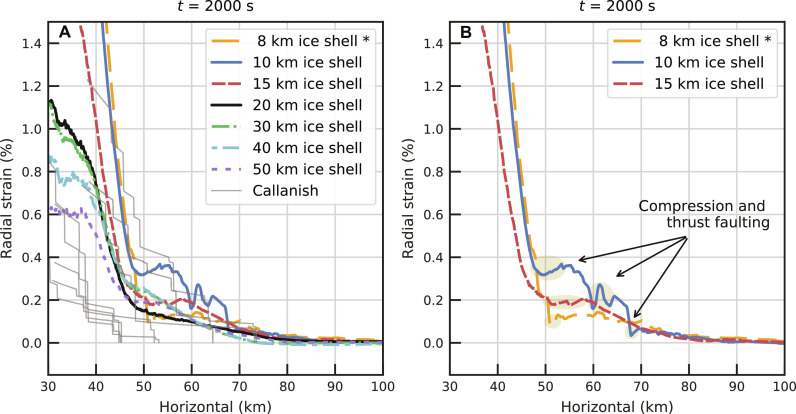
Effect of ice shell thickness on radial strain. Radial strain is calculated at an initial depth of 1 km (see details in Materials and Methods). The choice of initial depth has little effect on the results (fig. S11A). (**A**) Radial strain from simulations with an impactor radius of 1.5 km compared to observation of Callanish. Each thick colored line depicts the results of a simulation with a different ice shell thickness (see the legend). Thin gray lines in (A) are radial strain profiles at different azimuths determined from observed fault offsets of Callanish (*21*, *22*). Note that the 8-km ice shell only contains a conductive lid; all other simulations include a 6-km-thick conductive lid overlying convecting ice. Shaded region in (**B**) represents regions of compression and thrust faulting.

**Fig. 3.**
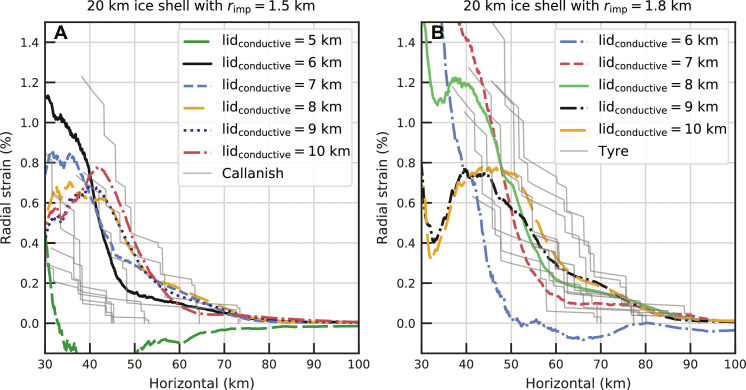
Effect of conductive lid thickness and impactor radius on radial strain. Same view as Fig. 2, but for various conductive lid thickness (lid_conductive_) and impactor radii (*r*_imp_) on a 20-km-thick ice shell (see the legend). (**A**) shows the results of simulations with a 1.5-km-radius impactor compared to observationally derived radial strain of Callanish, and (**B**) shows the simulation results with a 1.8-km-radius impactor compared to observationally derived radial strain of Tyre. Thin gray lines in (A) are radial strain profiles at different azimuths determined from observed fault offsets of Callanish and that in (B) for Tyre, respectively (*21*, *22*).

Radial strain estimates from observed fault offsets (*21*, *22*) allow us to make quantitative comparison between models and observations in the region 30 to 100 km from the basin center where graben occur (fig. S3). We calculate the radial strain in the models by tracking the radial displacement of tracer particles (Materials and Methods). Our simulations of 20- and 30-km-thick ice shells reproduce the observed radial strains well (Fig. 2A). The radial strain in our 20- and 30-km-thick ice shells are 0.8% at 40 km within the maximum strain of 0.6 to 1.2% measured for Callanish (Fig. 2A). Tyre is larger than Callanish, and 40 km from the basin center has a larger maximum radial strain of 1.0 to 1.5% (see Fig. 3B). Note that the observationally derived strain estimate at 40 km from the basin center (or any location) represents the total amount of radial strain experienced at that location from the movement of that surface inward toward the center of the basin from its original position. To determine this strain, the offset along all faults exterior to the point must be added (*21*, *22*). For purely extensional tectonics, as observed for Tyre and Callanish, the radial strain will monotonically increase as we move toward the basin center. For the 8-, 10-, and 15-km-thick ice shell simulations, the radial strain does not monotonically increase as we move toward the basin center (Fig. 2B). Local decreases in the radial strain represent compressive plastic strain and thrust faulting that occurs as the central uplift collapses and pushes material outward (figs. S5 and S6). Thus, an ice shell thinner than 15 km is inconsistent with observed outer rings of the basins. We note that Melosh and McKinnon (*24*) predicted that if the weak asthenosphere under the lithosphere is too fluid, oscillation of the crater floor will produce outward-going waves resulting in radial and concentric fracturing. Our simulations broadly validate the ring tectonic theory (*24*) including the conclusion that Valhalla-type multiring basins will not be produced if asthenosphere is too weak. For an ice shell 20 to 50 km thick, our results are similar except for a reduction in the radial strain 40 km from the basin center as the ice shell thickness increases. This similarity suggests that the ocean plays a limited role in the formation of icy multiring basins. Moreover, with our current observational constraints, ice shells 20 to 50 km thick are capable of reproducing the observed radial strains of Callanish, suggesting that these basins only provide a lower limit constraint on ice shell thickness. Valhalla-type multiring basins, however, require a relatively thin conductive ice shell overlying warm weak ice that can readily flow during crater collapse. This thermal structure is more likely to occur on icy bodies with warm interiors and subsurface oceans, so a connection between Valhalla-type multiring basins and ocean worlds is still expected despite the oceans’ limited role in Valhalla-type multiring basin formation.

### Impactor size and conductive lid thickness affect the radial strains

**Fig. 4.**
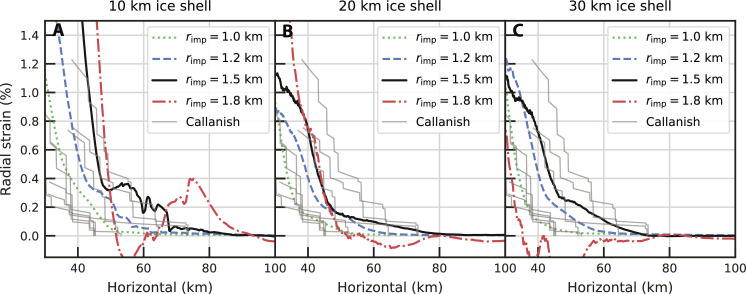
Effect of impactor radius and ice shell thickness on radial strain. Same view as Fig. 2, but for impactor radii (*r*_imp_) on the 10- to 30-km ice shell case. (**A**) shows simulation results with a 10-km-thick ice shell, (**B**) 20-km-thick ice shell, and (**C**) of 30-km-thick ice shell. Thin gray lines are radial strain profiles at different azimuths determined from observed fault offsets of Callanish (*21*, *22*). All simulations assume a 6-km-thick conductive lid overlying convecting ice.

Other than ice shell thickness, the parameters that have the greatest control on our simulations are impactor size and the thermal structure of the ice shell. First, we discuss the effects of impactor size for simulations with a 6-km-thick conductive lid. The radial strains produced by the 1.0-km-radius impactor are too small to explain observed basin rings (Fig. 4), and no obvious faulting occurs in our simulations (fig. S7). Although there are no clear zones of compression in the final radial strain for a 1.2-km-radius impactor on a 10-km-thick ice shell (Fig. 4A), we see thrust faulting occurring during the collapse of the central uplift (fig. S8), which is inconsistent with observations. The 1.2-km-radius impactor striking a 20- or 30-km-thick ice shell produces radial strains that are a reasonable fit for Callanish. However, we favor the larger 1.5-km-radius impactor simulations on the 20- to 30-km-thick ice shell, which produce slightly larger radial strains and nonzero radial strains extending further from the basin center than the 1.2-km-radius impactor simulations (Fig. 4, B and C). The lateral extent of heightened radial strain and faulting is more consistent with the observed rings of Callanish, which extend up to 75 km from the basin center. While a larger 1.8-km-radius impactor on the 20-km ice shell results in larger radial strains, the simulation also exhibits compression that is inconsistent with observations (Fig. 4B). Thus, we generally find that as we increase impactor size, the resultant radial strain increases in magnitude and lateral extent, and correspondingly, rings form further from the basin center. However, if the impactor size is too large, subsequent collapse of the larger central uplift is capable of pushing the ice shell outward, producing compressional features at locations inconsistent with observations (Fig. 4).

To isolate the effect of the ice shell’s thermal structure, we discuss the effect of conductive lid thickness while keeping the impactor radius and ice shell thickness fixed at 1.5 and 20 km, respectively. When we consider a 5-km-thick conductive lid, the ice shell is too weak, and the collapse of the central uplift produces strong compression in the region where rings are observed, which is inconsistent with observations. A 6- to 7-km-thick conductive ice lid on top of convecting ice at 265 K, however, reproduces the observed radial strain of Callanish well (Fig. 3A). This is consistent with the preferred thermal structure found by Silber and Johnson (*17*) based on the morphology of smaller craters. When the conductive lid is too thick (≥8 km), and the ice shell is too strong, a region of strong compression occurs inward of about 45 km from the basin center (Fig. 3A). We disfavor these models as there are no clearly compressional features observed in the outer ring region of Tyre or Callanish (fig. S3). In addition, for these thicker conductive lids, we do not see localized features consistent with ring formation (fig. S9). For these stronger ice shells, the collapse of the central uplift causes compression close to the basin center (inward of 40 km) rather than simply reducing the magnitude of extension as in the case of the 6-km-thick conductive lid. This explains how these models maintain similar radial strain outside of 40 km from the basin center despite the thicker conductive lids not leading to ring formation. Thus, we prefer a 6-km conductive lid, which produces localized ring-like features and is in agreement with previous estimates of thermal structure needed to reproduce the morphology of smaller Europan craters (*17*).

Our simulations suggest that Europa’s ice shell is composed of a conductive lid over a warm convecting sublayer. Silber and Johnson (*17*) suggested that the fully conductive ice shell over the ocean can also explain the transitions in depth-to-diameter ratios of Europa’s craters. However, our simulations of impacts into a purely conductive 8-km-thick ice shell (with an ocean under it) result in compressional strain and thrust faulting similar to the 10- and 15-km ice shell cases; decreases in the radial strain indicate regions of compression (Fig. 2B). Thus, our simulations clearly favor an ice shell that is at least 20 km thick and composed of a 6-km-thick conductive ice lid over warm convecting ice for Callanish. The choice of the convecting temperature also provides another effect on the formation of multiring basins. The colder convective temperature of 255 K results in a larger yield strength within the convective sublayer (fig. S10). As a result, the colder convective temperature results in less inward flow and lower radial strain (fig. S11C). We investigated many other parameters to ensure that our results are not affected by small uncertainties in the choice of input parameters. Most of the parameters we varied have minor effects on our results except for the frictional coefficient for damaged material and the maximum dilatancy coefficient (Supplementary Materials and figs. S11 and S12).

### A larger impactor and thicker conductive lid for Tyre

With an estimated equivalent diameter of 38 km, Tyre is somewhat larger than Callanish [33-km diameter (*21*, *22*)]. In addition, the radial strain inferred from observations of graben surrounding Tyre implies a higher radial strain than for Callanish. We find that these larger radial strains are the result of a larger impactor with a 1.8-km radius compared to 1.5 km for our fiducial simulation. While a 1.8-km-radius impactor on a 6-km-thick conductive lid exhibits compression (Fig. 3A), a 1.8-km-radius impactor on an 8-km-thick conductive lid reproduced the observed radial strain of Tyre well (Fig. 3B). Localized ring-like structures also form in this simulation (fig. S13), which cannot be seen in the case of the 1.5-km-radius impactor with the same 8-km-thick conductive lid (fig. S9). The slight difference in conductive lid thickness between our best fit for Tyre (8-km-thick conductive lid) and Callanish (6-km-thick conductive lid) could be the result of spatial or temporal variation in Europa’s heat flow. Note that a 7-km-thick conductive lid produces marginal fits for both cases, so a similar conductive lid thickness for Tyre and Callanish cannot be ruled out. When the conductive lid gets thicker (≥9 km) in our runs with a 1.8-km-radius impactor, compression occurs close to the basin center (Fig. 3B). Again, this is inconsistent with the observations that do not find any morphological signs of compression in the graben region [(*21*, *22*), see also Discussion]. These trends are similar to those seen for the 1.5-km-radius impactors (Figs. 3A and 4B). Overall, a 1.8-km-radius impactor striking an ice shell that is at least 20 km thick with an 8-km-thick conductive lid is our best fit model for Tyre.

## DISCUSSION

Our simulations indicate that an ice shell thicker than 20 km composed of a conductive lid overlying a warm convecting sublayer is required to reproduce the observed structures of Europa’s multiring basins. We can also consider the post-impact structure and any associated ice-shell ocean uplift that may be observed by future spacecraft missions. In the case of our thin ice shells (10 and 15 km), there is a large uplift of ice-ocean interface (figs. S5D and S6D). Through isostasy, this uplift produces a basin that is 0.5 km deep, deeper than observed [<0.1 km, (*9*)]. This uplift would need to viscoelastically relax for the basins to match present-day topography. In contrast, uplift of the ice-ocean interface is almost absent in the 20-km ice shell case (fig. S2). The efficient inward flow of the warm weak portion of the ice shell, required for multiring basin formation, results in a final ice shell thickness that is nearly unperturbed after impact. This results in a final crater floor with negligible topography. Thus, our best fitting models require limited post-impact viscoelastic relaxation to match observed topography. In all cases, the center of the basin contains a melt pool (fig. S2B), which may cause exchange between the surface and ocean even in the case of a thick ice shell (*38*).

Our results show that there is some trade-off between impactor size and lithospheric thickness. We find that a larger impactor with a thicker lithosphere and a smaller impact into a thinner lithosphere can produce similar results (Fig. 3). We also find that larger impactors generally result in larger radial strains and rings forming further from the basin center. This result is broadly consistent with the scaling put forward by Melosh (*39*) that the extent of basin rings is proportional to the transient crater size but inversely proportional to the lithosphere thickness. However, if the impactor is too large, we find that collapse of the central uplift, which was not accounted for in (*39*), results in large regions of compression (Fig. 4). Thus, the scaling in (*39*) should be used with caution.

We have focused on the outer ring region of multiring basins where extensional tectonics (i.e., graben) are observed, and we have quantitative constraints on the radial strain. In the inner ring region of Tyre and Callanish, annular massifs/ridges exist (*19*, *20*). We should mention that the massifs or ridges usually imply compressional tectonics; however, the inner ridges of Tyre and Callanish appear to be tilted blocks formed via breakup or extension (*21*, *22*). No previous work hypothesizes the inner ridges as a consequence of compression (*18*, *19*, *40*, *41*), except for a tentative suggestion in (*20*). Nevertheless, we cannot rule out the possibility that there is nonobvious or cryptic compression in the inner region. Our simulations with thicker conductive lids show decreases in the radial strain 30 to 40 km from the basin center, which is indicative of compressional tectonics (7- to 10-km conductive lid thickness in Fig. 3A and 8- to 10-km conductive lid thickness in Fig. 3B). These same simulations have extensional features and radial strains that agree with observations in the outer ring region. Thus, if the inner ring region is indicative of compression, simulations with ice shells thicker than 20 km can explain compression in the inner ring region and extension in the outer ring region. In contrast, in the thin ice shell, the opposite tectonic pattern occurs. Thin ice shell simulations exhibit extension at 30 to 40 km from the basin and compression beyond that (Fig. 2B), which is inconsistent with observations.

Lateral variations of Europa’s ice shell thickness are thought to be limited on a global scale (*42*). Thus, it is reasonable to extrapolate the ice shell thickness at one location to Europa globally. It has been suggested that Europa’s ice shell thickness may vary substantially over time though (*9*). While Europa’s average surface age is estimated to be only 20 to 200 Ma (*43*), it is unlikely that both Tyre and Callanish have similar ages. We find that Tyre is best fit by an impact into a target with a slightly thicker, 8-km-thick, conductive lid, which may indicate spatial or temporal variation in Europa’s heat flow. Both basins, however, require an ice shell thicker than approximately 20 km at the time of their formation. This suggests that any temporal thickness variations are unlikely to reduce the ice shell thickness below 20 km during the present era. An ice shell thicker than 20 km should consist of a conductive lid over a convecting sublayer (*44*). This agrees with our constraints on a minimum shell thickness of 20 km that have a conductive ice lid over the convective ice layer. The 20-km-thick ice shell is also consistent with the estimate based on the diameter at which the craters become shallow and exhibit multiring structures [19 to 25 km ice shell thickness; Schenk (*14*)]. However, our study suggests that icy multiring basin formation provides constraints on the minimum thickness of the ice shells they form on. Therefore, Europa’s ice shell could be thicker than 20 km, and the ice shells of Ganymede and Callisto may be substantially thicker than 80 to 105 km, as suggested in (*14*).

## MATERIALS AND METHODS

### Numerical methods

To examine multiring basin formation on Europa, we perform impact simulations using the iSALE-2D shock physics code (*30*, *31*, *45*). The iSALE code has been developed to model planetary impacts and cratering and improved from the SALE code (*29*), by including various equations of state and a strength model (*31*, *46*, *47*). We assume that a spherical icy impactor hits a flat target consisting of an ice shell over a subsurface ocean. We use Tillotson Equation of State (EOS) (*48*, *49*) for icy materials and ANEOS for the water ocean (*13*). The impactor size ranges from 1.0 to 1.8 km in radius, which corresponds to the high end of Jupiter Family comet sizes (*50*, *51*). Since we use the same EOS for the target and impactor, their density depends on temperature and pressure; their reference density is 910 kg/m^3^ (*15*). We use the strength model parameter from Bray (*15*, *52*), which fits to laboratory measurements of intact (*53*) and damaged ice (*54*). To approximate the effects of moderately oblique impacts, the impact velocity for an axisymmetric simulation is often taken to be the vertical component of an oblique impact. Our choice of 15 km/s corresponds to the vertical component of an oblique impact velocity of 26 km/s with the impact angle of ~35°. Simulating vertical impacts at 15 km/s also reduces computational expense and maintains consistency with previous work (*15*–*17*). Although we only explore the different impactor sizes, variation in impact velocity would have a similar effect on our results (*23*, *35*). To resolve the graben within a multiring basin, we use a resolution of 50 m [the domain size of a high-resolution zone in iSALE is 100 km in the horizontal (radial) direction and 5 km in the vertical direction]. Note that our computational mesh region is large enough to prevent reflections that may affect the results (an extension zone in iSALE covers up to ~800 km in the horizontal direction and from about −400 to ~140 km in the vertical direction).

We treat ice shell thickness on Europa as a free parameter and simulate impacts into ice shells that are 8, 10, 15, 20, 30, 40, and 50 km thick. We assume that the surface temperature of the target is 100 K. The temperature profile in the target is one of the primary controls of basin formation (*17*, *27*, *28*). We consider an ice shell composed of a 6-km-thick conductive ice lid over a convecting sublayer with a temperature of 265 K as our fiducial case. The temperature gradient within the conductive ice lid is 28.85 K/km. This is one of the ice shell structures that can explain the depth-to-diameter ratio of smaller craters on Europa (*17*). To test the effect of temperature profile, we varied conductive lid thickness from 5 to 10 km thick from our fiducial case with a 6-km-thick conductive lid. We also consider targets with a convective temperature of 255 K and conductive lid thicknesses of 6 km, which are also found to reproduce the morphology of smaller craters (*17*). We also simulate impacts into a fully conductive ice shell that is 8 km thick, which can also explain the morphology of smaller Europan craters (*17*). On the basis of previous work (*26*, *55*), we include a viscoelastic-plastic ice rheology in our simulations and include the dilatancy model following (*33*). Our input parameters of materials are summarized in table S1.

To evaluate agreement with observations, we calculate the radial strain using the radial displacement of tracer particles. The Lagrangian tracer particles in iSALE simulations track the movement of parcels of material through an Eulerian grid during the crater formation. Since cells in the near surface layer may be overly affected by ejecta emplacement, we consider the radial displacement at the initial depth of ~1 km. When we explore the dependence of initial depth (0.5 to 1.5 km) on the radial strain, we confirmed that the choice of initial depth in the shell does not affect the radial strain profile of our fiducial case at distances greater than 40 km from the basin center (fig. S11A). On the other hand, if we consider radial strain at greater depth (3.0 and 4.0 km), the radial strains become smaller and are no longer representative of the deformation we observe at the surface. Graben are observed from around 40- to 80-km horizontal distance from the basin center of Tyre and Callanish (fig. S3) (*21*, *22*). We note that the definition of basin center is better for Tyre because the entire basin was imaged at moderate resolution. Only about half of the Callanish basin was imaged at moderate-to-high resolution, which may cause some uncertainty in the center fit and the offset in radial strain (*21*, *22*). Following the work of Singer *et al.* (*21*, *22*), which describes the radial strain of the observation, we accumulate the radial displacement from 100 km toward the center as ΣΔ*r*/(*r* + ΣΔ*r*), where *r* is the radial distance, and Δ*r* is the radial displacement; thus, ΣΔ*r* represents the total inward displacement, and the denominator (*r* + ΣΔ*r*) indicates the initial distance. In our numerical simulations, we consider all tracer particles at the given initial depth to calculate the radial strain. As the passage of shock wave finishes at 50 s after the impact, we take this as a radial distance of tracer particles at that time [i.e., *r*(*t* = 50)]. The total inward displacement is given by ΣΔ*r* = *r*(*t* = 50) − *r*(*t* = 2000), where positive values indicate inward displacement. Note that we calculate inward displacement of tracer particles regardless of total plastic strain they experience. The smooth variation of our calculated radial strain is another indication that the radial strains in our simulations are not as localized as in reality. Variation in observed radial strain may come from natural variation or from inaccuracies in determining the basin center with available Galileo images in the case of Callanish. Hence, we compare the observationally derived strain data and our numerical simulation in the horizontal direction. Note that the observed graben have widths of 0.4 to 4.7 km and depths of 14 to 210 m (*21*, *22*). At least 20-m resolution is required to resolve these depths. Although our resolution of 50 m is not enough to resolve the observed depths, our results demonstrate that it is sufficient to reproduce the observed radial strain.

## Supplementary Materials

### This PDF file includes:

Supplementary Text Figs. S1 to S13 Table S1 Legend for movie S1 References
