# Supplementary material for: Multiring basin formation constrains Europa’s ice shell thickness
Source: Sci Adv. 2024 Mar 20;10(12):eadj8455. doi: 10.1126/sciadv.adj8455 (PMC10954210; doi:10.1126/sciadv.adj8455)
Supplement: Supplementary file 1 — Supplementary Text Figs. S1 to S13 Table S1 Legend for movie S1 References [file sciadv.adj8455_sm.pdf]

Science Advances
AAAS

Supplementary Materials for

Multiring basin formation constrains Europa's ice shell thickness

Shigeru Wakita *et al.*

Corresponding author: Shigeru Wakita, shigeru@mit.edu

Sci. Adv. **10**, eadj8455 (2024)
DOI: 10.1126/sciadv.adj8455

The PDF file includes:

Supplementary Text
Figs. S1 to S13
Table S1
Legend for movie S1
References

Other Supplementary Material for this manuscript includes the following:

Movies S1

Supplementary Text

Effect of maximum dilatancy coefficients

The choice of the maximum dilatancy coefficient (β) affects the radial strain significantly. The dilatancy coefficient takes its maximum value (β) at zero porosity, pressure, and temperature, it decreases according to the increases of those parameters (33). A dilatancy angle of 4–5 degrees appropriate for ice (56, 57) corresponds to a dilatancy coefficient of β =0.07–0.09. While we used the fiducial value of 0.09, we use β = 0.045 and 0.18 to explore how β affects the radial strain. This is the same range considered by (1) who explored the effect of dilatancy on the formation of terrestrial craters. In the case of β = 0.18, the radial strain is lower than our fiducial simulation with β = 0.09 (Fig. S11B). In the simulation with of β = 0.045, however, the radial strain is higher than our fiducial model, resulting in strains that are still in agreement with observations.

Effect of other material coefficients

Another influential parameter is the damaged frictional coefficient (μ_d). As this value gets lower, the material strength decreases. Following previous work on icy satellites (15, 17), we took the fiducial value of μ_d = 0.6. While the case of μ_d = 0.7 results in a similar radial strain profile as the fiducial case, the weaker case of μ_d = 0.5 is too weak and results in compression (Fig. S11D). Note that there are likely tradeoffs between thermal structure and material coefficients for example a μ_d = 0.5 could work if a colder ice shell structure is considered. Although there are other coefficients (μ_i, μ_c) in the iSALE material parameters (Table S1), they have a very weak effect on the radial strain profile (Figs. S11E, S11F).

Effect of damage model, acoustic fluidization, and tensile failure

In our fiducial run, we used the IVANOV damage model in iSALE code (46). We explored the effect of the damage model by using the COLLINS damage model (30), but found that the choice of the damage model has minor effect on the radial strain (Fig. S12A). In our fiducial model, we include the effect of acoustic fluidization, which models the transient weakening of the target during the crater-forming impacts (58). We ran a simulation without acoustic fluidization to test its effect on our results. While acoustic fluidization reduces the material strength out to ~55 km from the center, we find that the acoustic fluidization model has a slight effect on the radial strain beyond 40 km from the center (i.e., the region of graben, Fig. S12B). When we ignore tensile failure, however we find a resulting decrease in the radial strain (Fig. S12C). Thus, considering tensile failure is important when simulating the formation of the multiring basins as tensile failure can weaken material far from the point of impact.

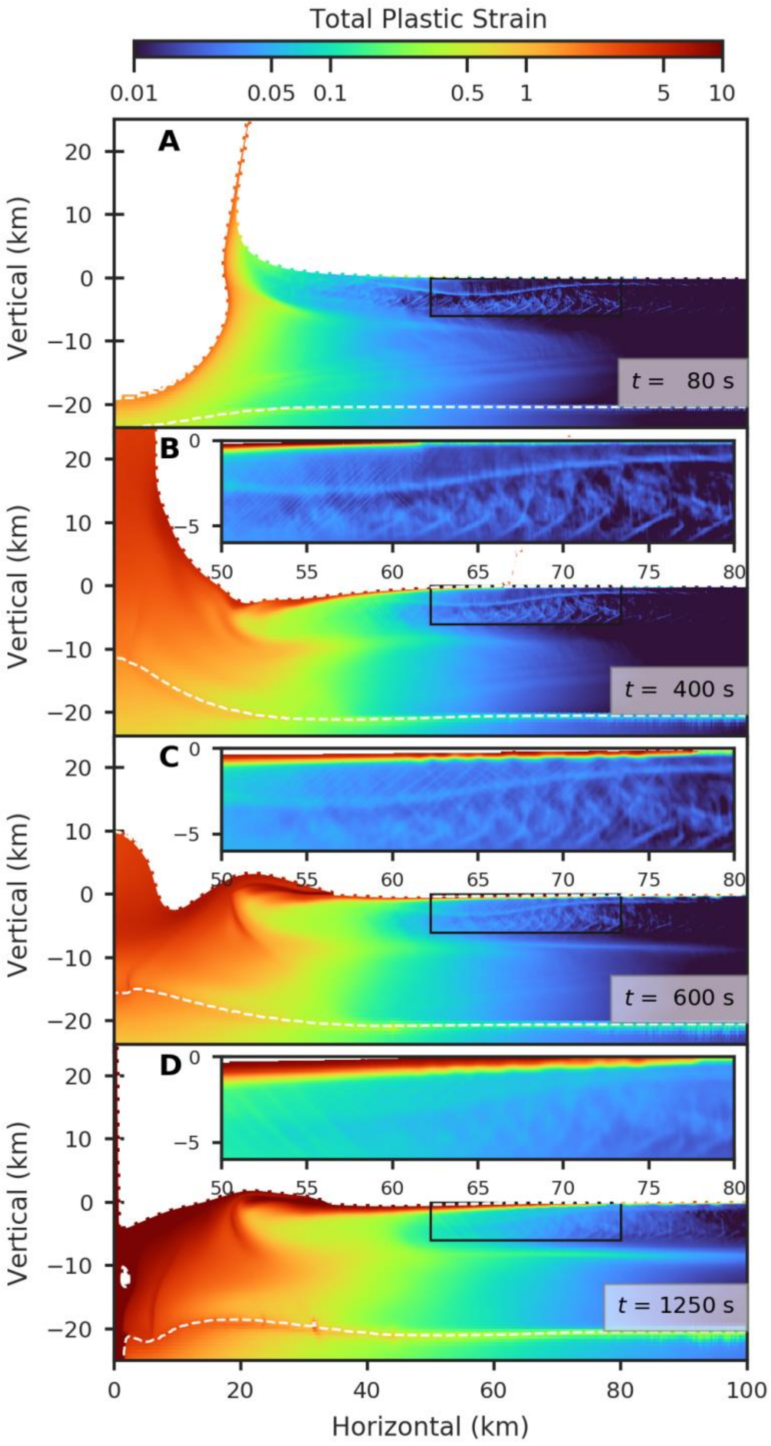

Fig. S1. Distribution of total plastic strain. Same viewing as Fig. 1, but this illustrates the total plastic strain in log scale.

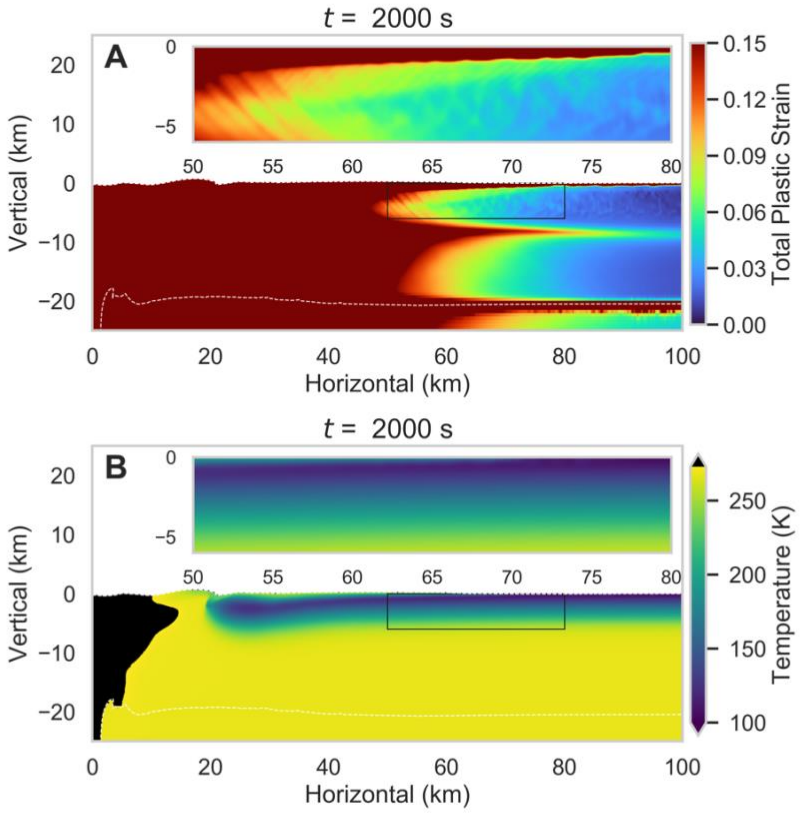

Fig. S2. Distribution of total plastic strain and temperature. (A) Same viewing as Fig. 1, but the snapshot at 2000 s after the impact. (B) Same model as Fig. 1, but the color indicates the temperature. Black color illustrates the temperature above 273 K, suggesting the presence of melt pool.

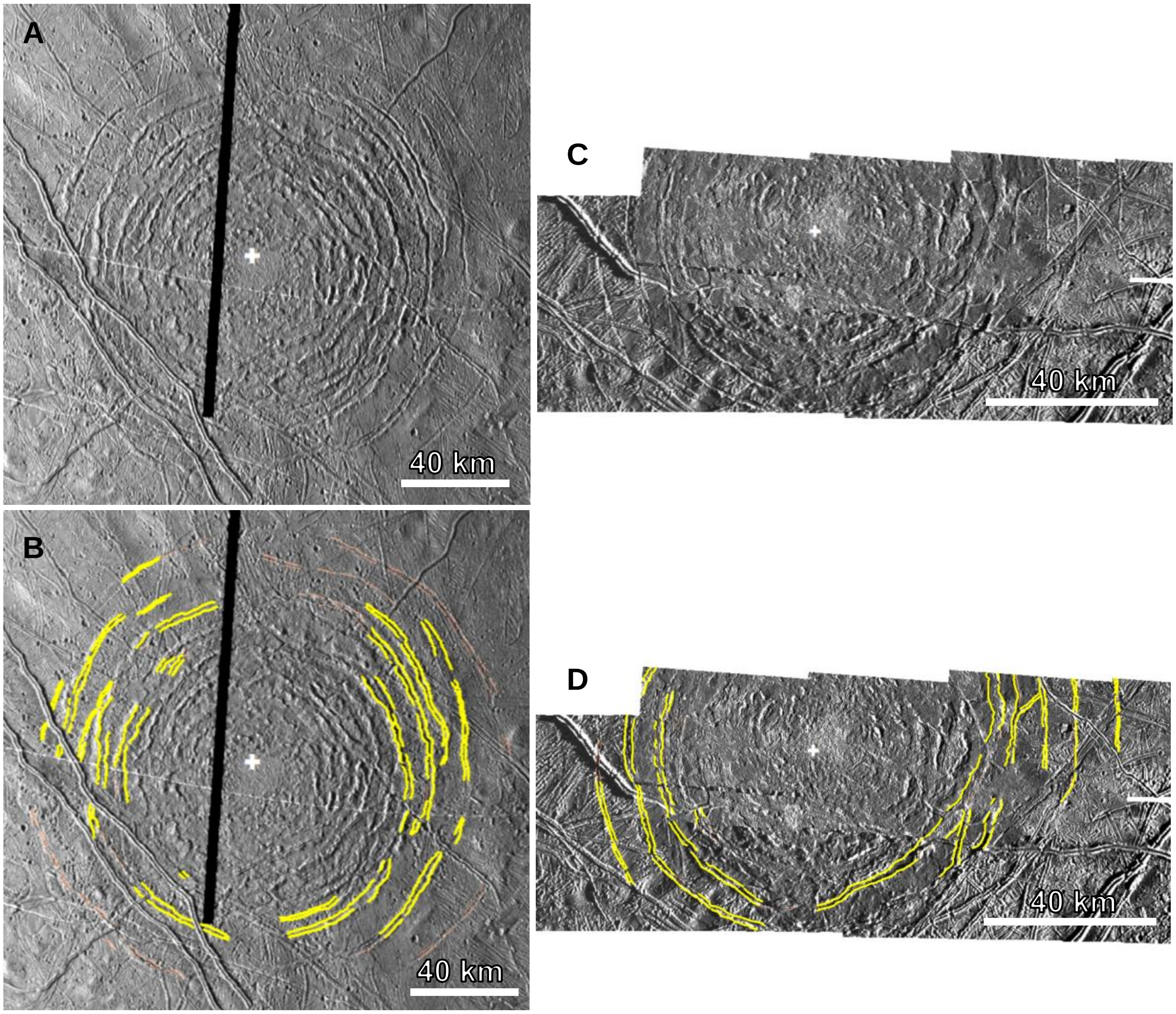

Fig S3. Tyre (panels A and B) and Callanish (panels C and D) as viewed from Galileo with mapped outlines of graben in lower panels (modified from Singer et al. (22)). Solid yellow graben outlines indicate more distinct graben, while lighter orange, dashed outlines indicate shallower or less distinct graben. The white plus signs indicate the average center point from fitting circles to the graben outlines for each basin.

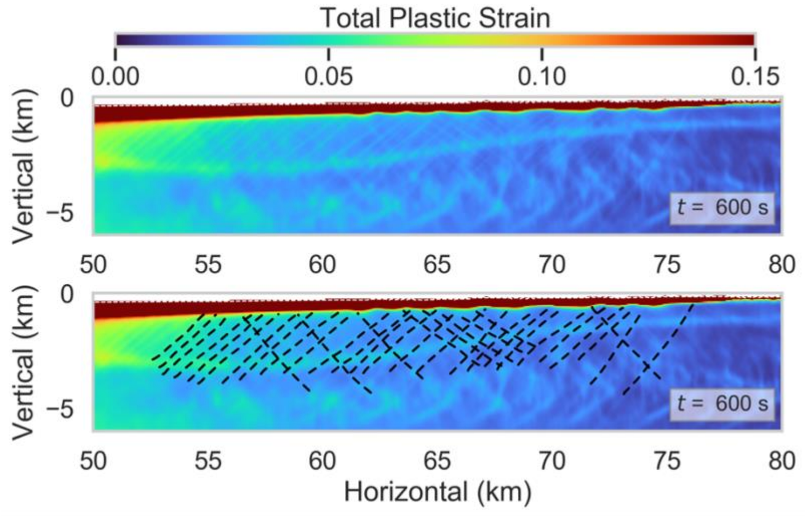

Fig. S4. Distribution of total plastic strain. Same viewing as the inset of Fig. 1C (top panel), but the bottom panel also outlines the location of faults as black dashed lines.

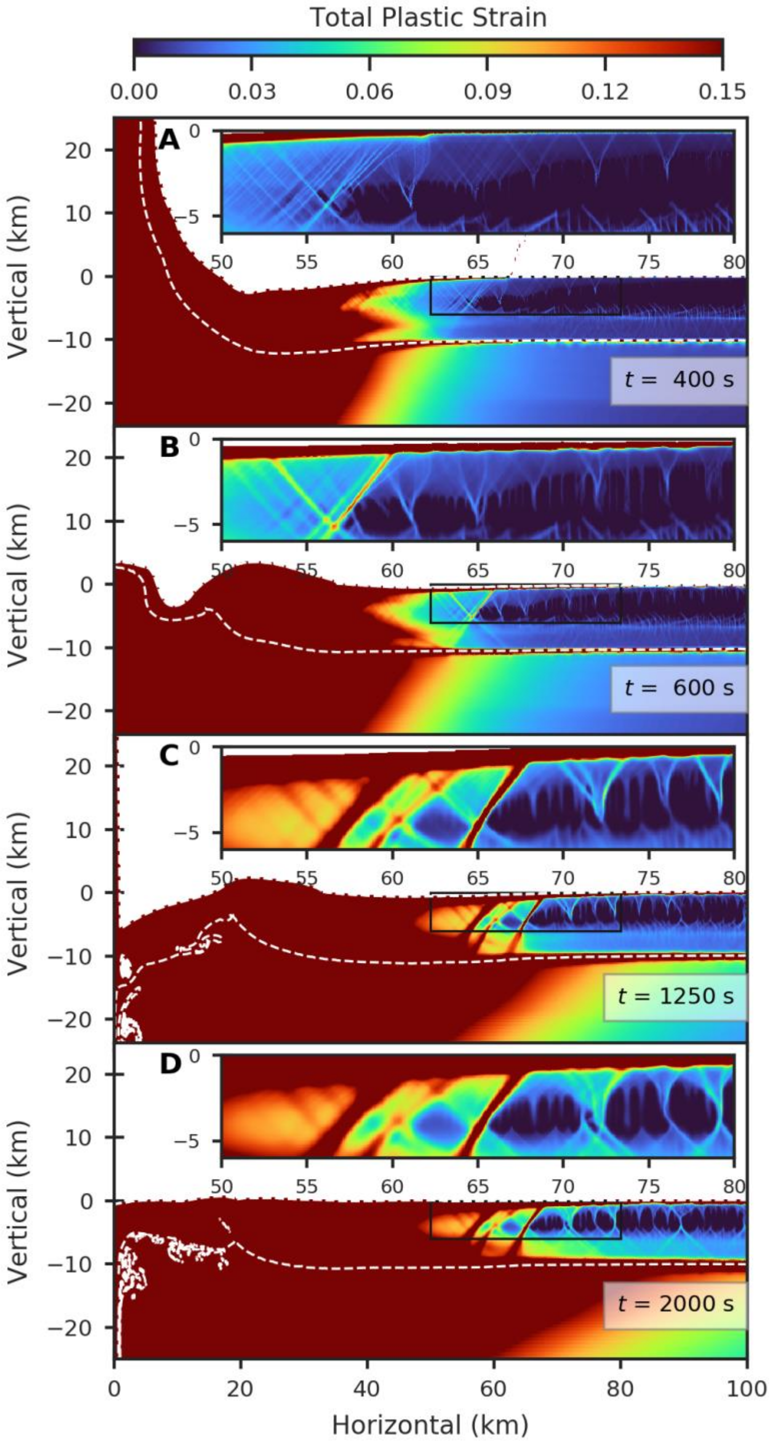

Fig. S5. Distribution of total plastic strain. Same viewing as Fig. 1, but with 10 km thick ice shell. Simulation has the same 1.5 km radius impactor and 6 km thick conductive lid used in Fig. 1.

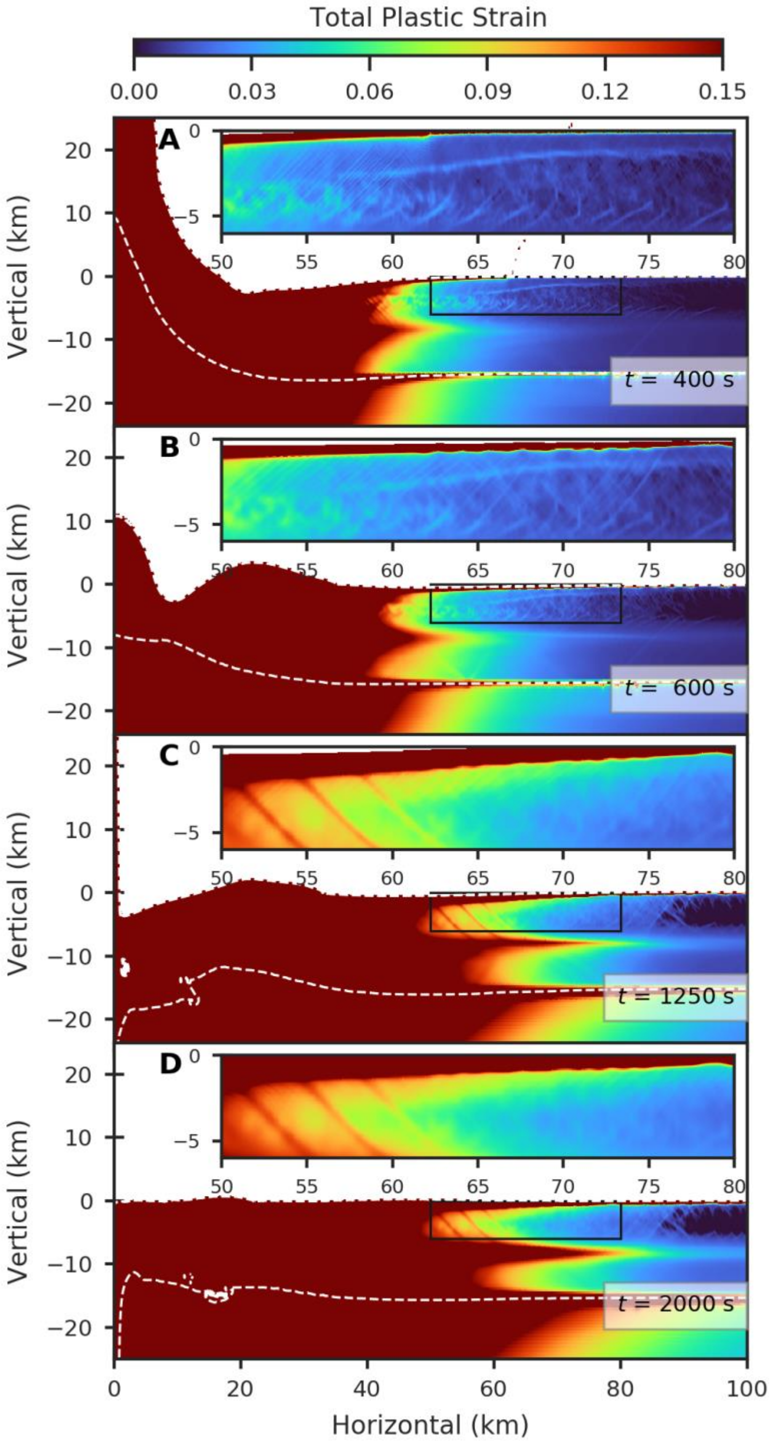

Fig. S6. Distribution of total plastic strain. Same viewing as Fig. 1, but with 15 km thick ice shell. Simulation has the same 1.5 km radius impactor and 6 km thick conductive lid used in Fig. 1.

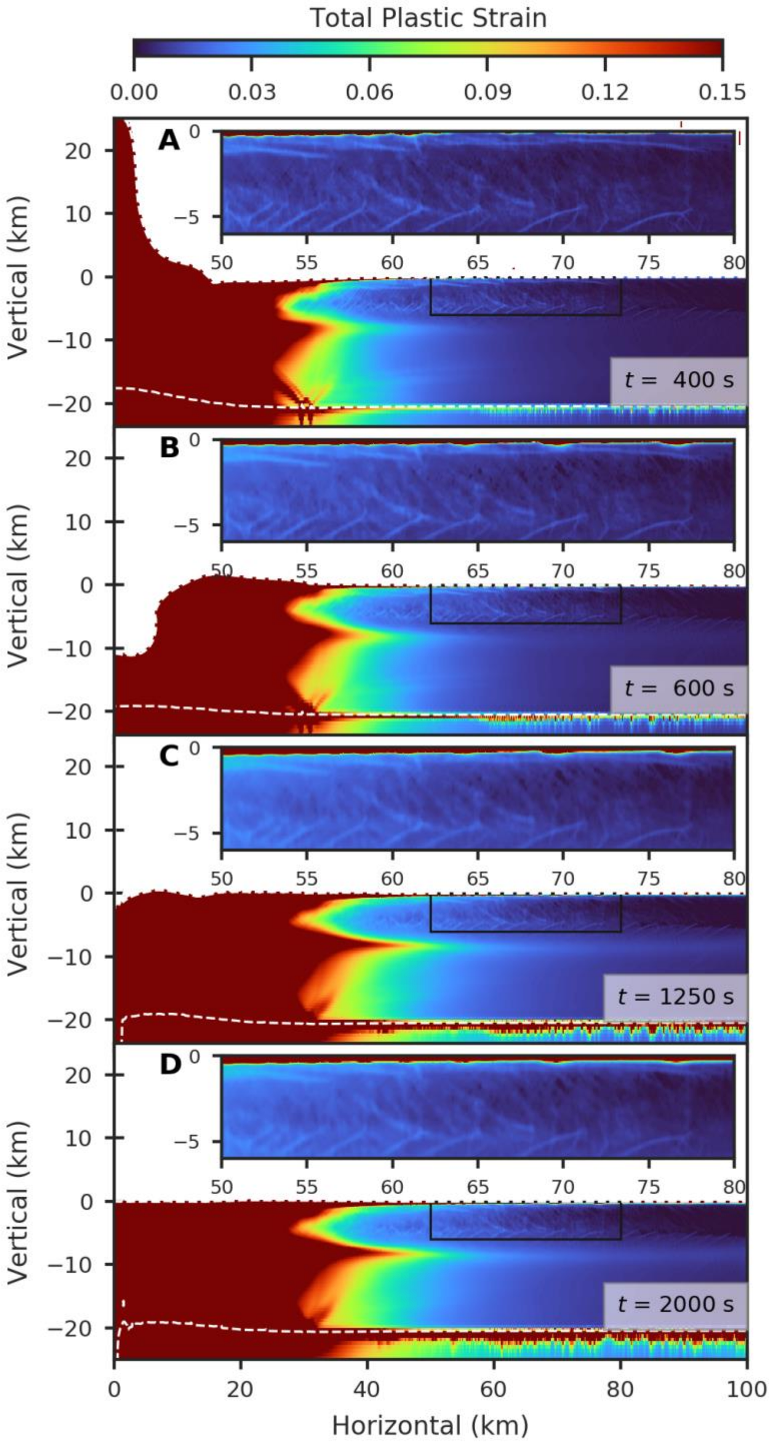

Fig. S7. Distribution of total plastic strain. Same viewing as Fig. 1, but for a 1.0-km-radius impactor. Simulation has the same 20 km thick ice shell and 6 km thick conductive lid used in Fig. 1. There is no clear faulting (see Fig. 4B).

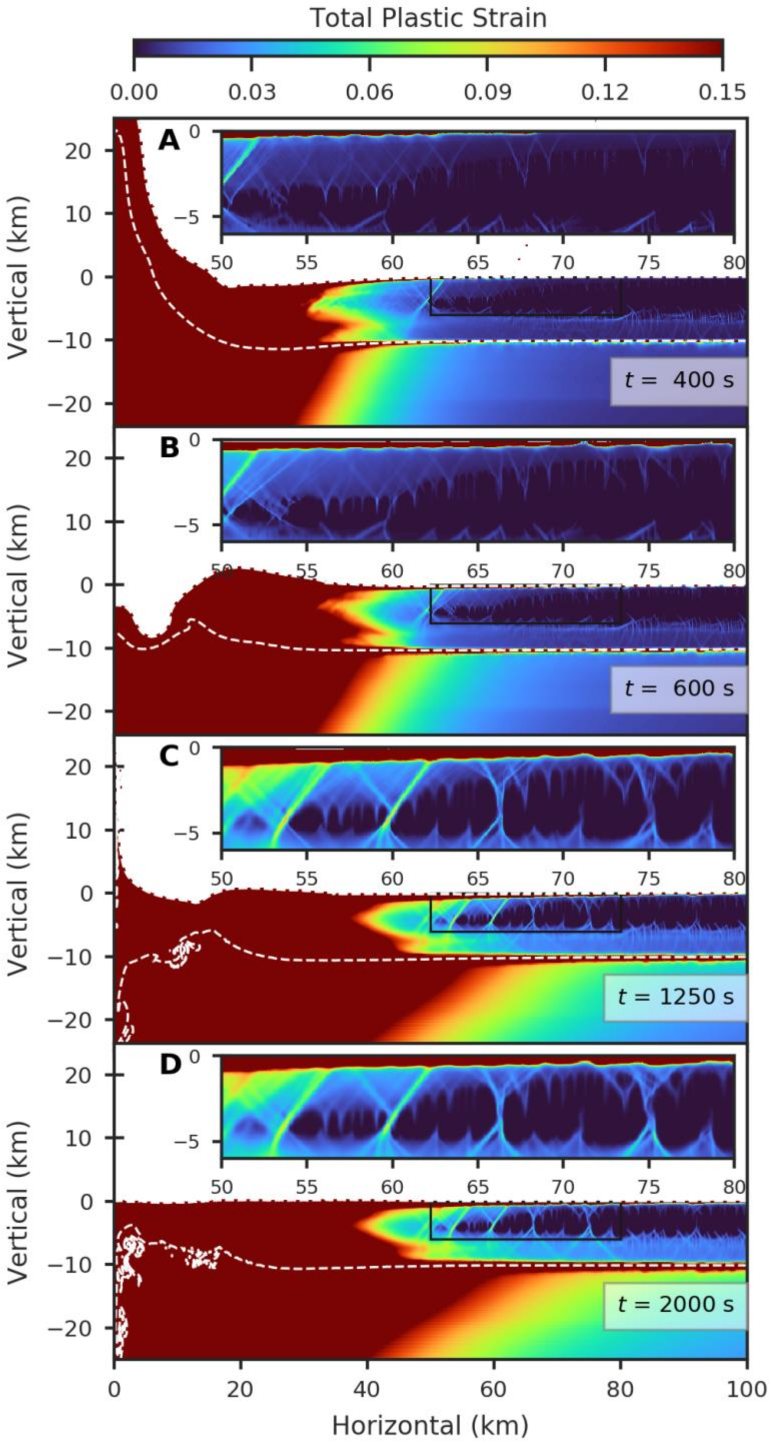

Fig. S8. Distribution of total plastic strain. Same viewing as Fig. 1, but for a 1.2-km-radius impactor and 10 km thick ice shell. Simulation has the same 6 km thick conductive lid used in Fig. 1.

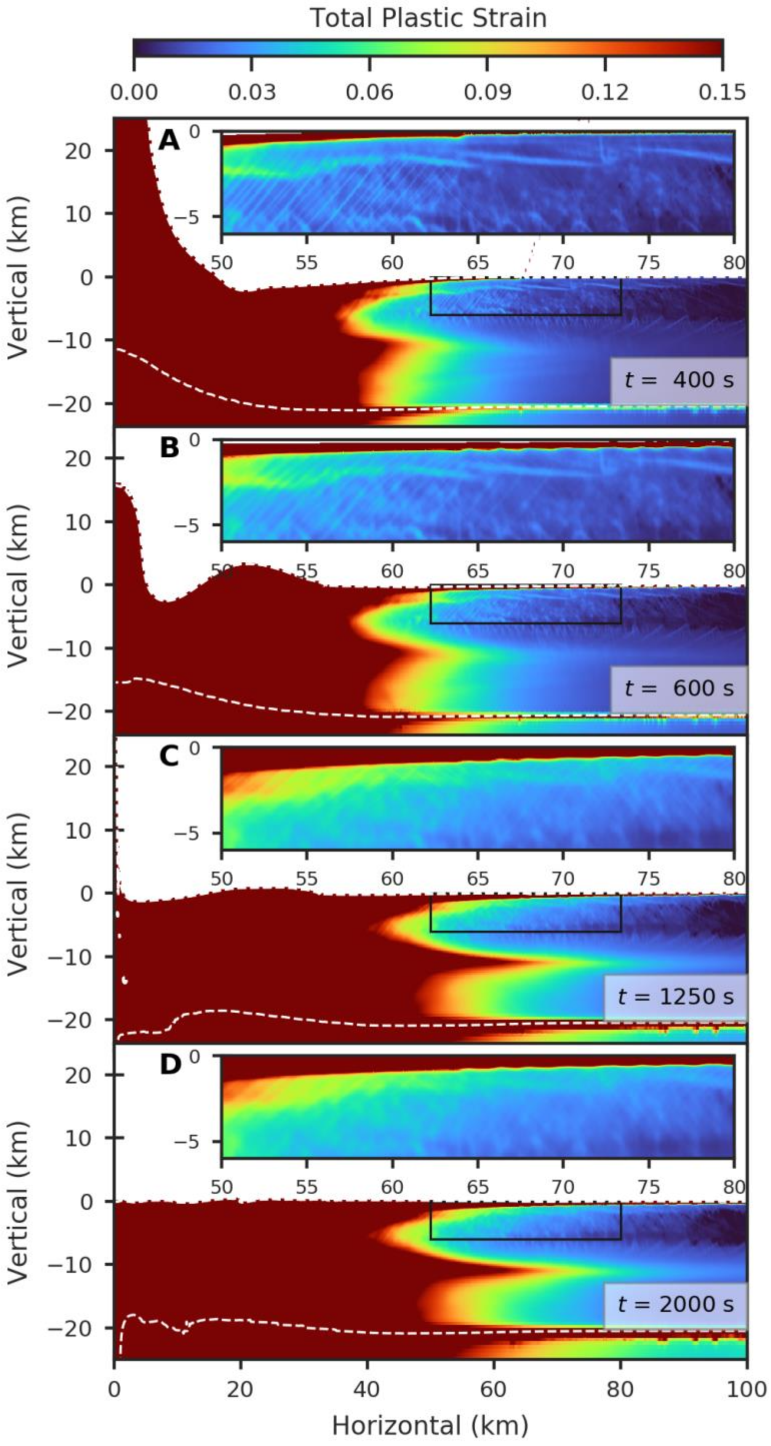

Fig. S9. Distribution of total plastic strain. Same viewing as Fig. 1, but for an 8 km thick conductive lid. Simulation has the same 1.5 km radius impactor and 20 km thick ice shell used in Fig. 1. There is no clear faulting (see Fig. 3A).

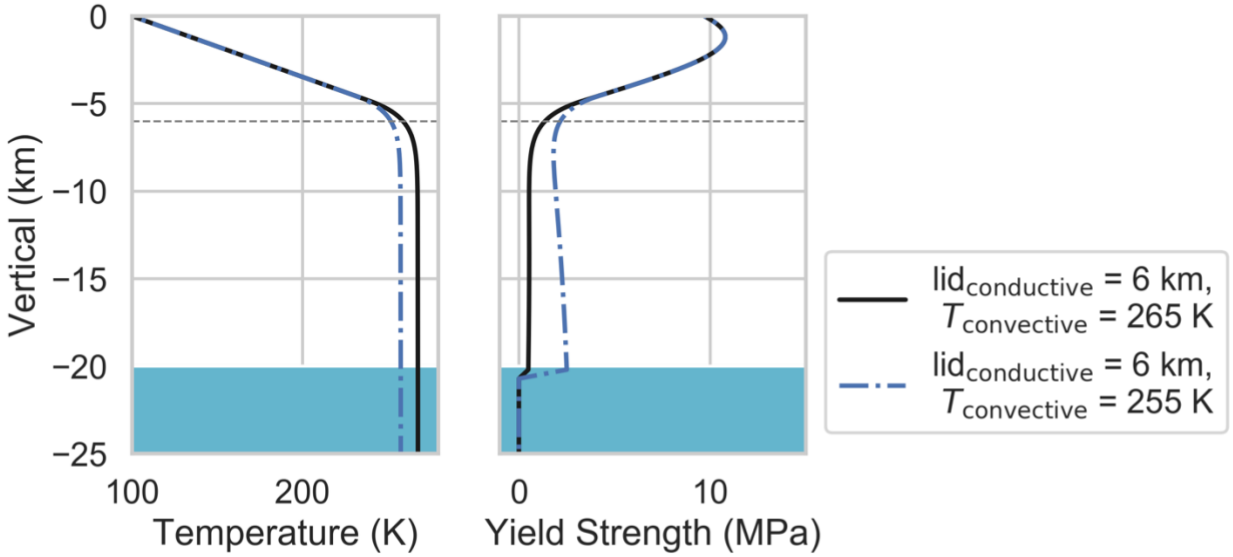

Fig. S10. Profile of temperature and yield strength in the ice shell. Each line depicts the different temperatures of convecting ice (see legend). The horizontal dotted lines indicate the bottom of the conductive lid (6 km), and the shaded region illustrates the ocean.

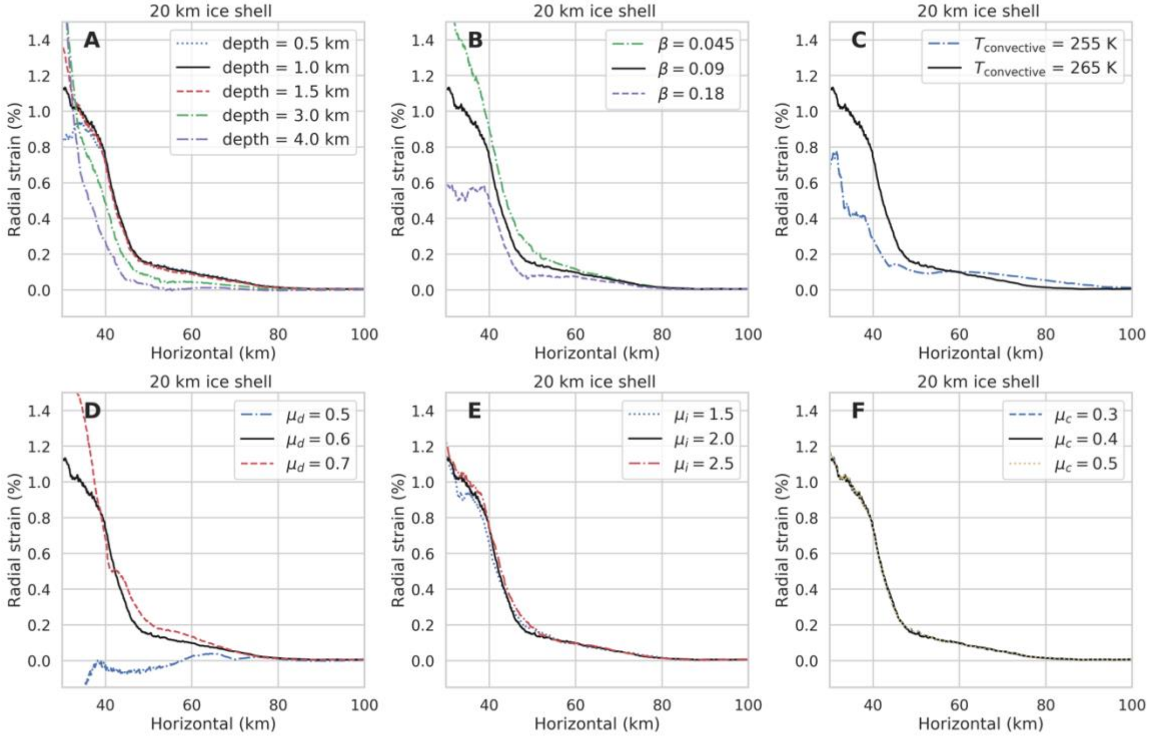

Fig. S11. Effect of parameters on radial strain. Same viewing as Fig. 2, but for various parameters in the 20 km thick ice shell. The black solid lines in each panel represent our fiducial case; 1.5-km-radius impactor, β = 0.09, the initial depth of 1 km, 6 km conductive lid with the convecting temperature of 265 K, μ_d = 0.6, μ_i = 2.0, and, μ_c = 0.4. Panel (A) depicts the initial depth, (B) for the maximum dilatancy coefficient (β), (C) for the convecting temperature, (D) for the damaged frictional coefficient (μ_d), (E) for the frictional coefficient of intact rock (μ_i), and (F) for the critical friction coefficient (μ_c), respectively.

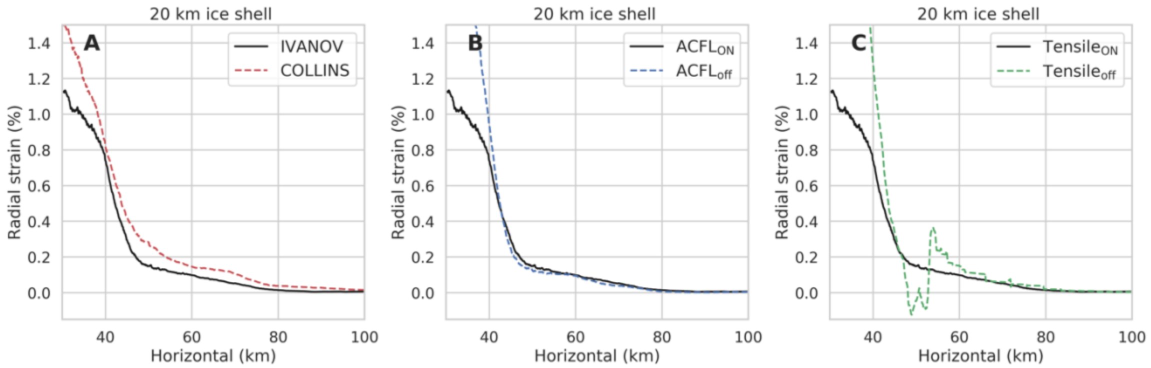

Fig. S12. Effect of models on radial strain. Same viewing as Fig. 2, but for various models in 20 km ice shell case. The black solid lines in each panel represent our fiducial case. Panel (A) shows the choice of damage model (IVANOV and COLLINS), (B) shows the effect of the considering/ignoring acoustic fluidization (ACFL), and (C) shows the effect of considering/ignoring the tensile failure.

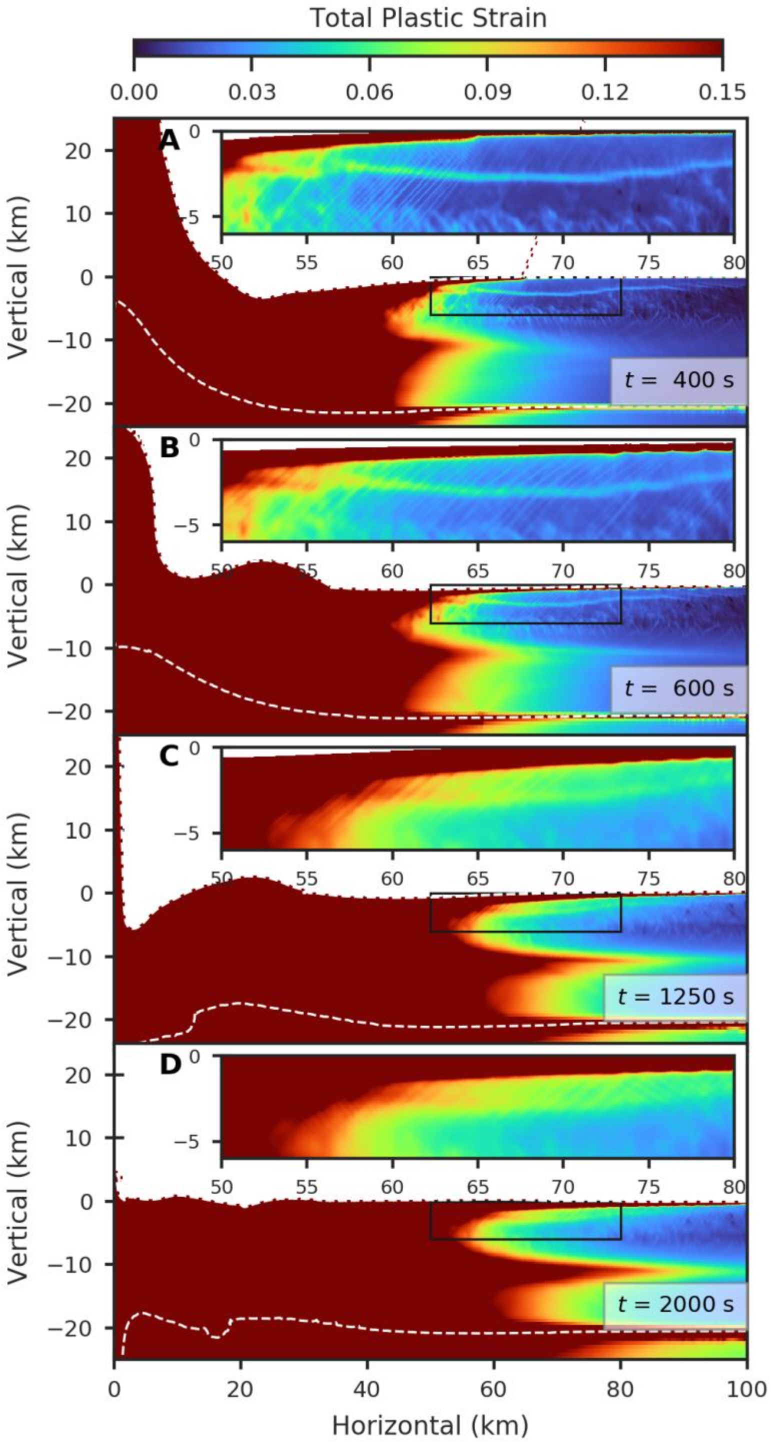

Fig. S13. Distribution of total plastic strain. Same viewing as Fig. 1, but for an 8 km thick conductive lid with a 1.8-km-radius impactor. Simulation uses the same 20 km thick ice shell as in Fig. 1.

Table S1. iSALE material input parameters for a fiducial case.

Description	Value
Equation of state	Tillotson, H_2O (*48, 49*)
Thermal softening parameter	1.2 (*15*)
Cohesion (damaged)	0.01 MPa (*15*)
Cohesion (undamaged)	10 MPa (*15*)
Frictional coefficient (damaged) [μ_d]	0.6 (*17*)
Frictional coefficient (undamaged) [μ_i]	2 (*15*)
Strength at infinite pressure	0.11 GPa (*15*)
Maximum dilatancy coefficient [β]	0.09 (*33*)
Dilatancy pressure limit	50 MPa (*33*)
Critical distension	1.2 (*33*)
Critical friction coefficient [μ_c]	0.4 (*33*)
Tensile strength (undamaged)	0.17 MPa (*33*)
Damage model	IVANOV (*46*)

Movie S1 (separate file). An animation of an icy multiring formation. Same viewing of Fig. 1; material is colored according to total plastic strain.

REFERENCES AND NOTES

1. K. K. Khurana, M. G. Kivelson, D. J. Stevenson, G. Schubert, C. T. Russell, R. J. Walker, C. Polanskey, Induced magnetic fields as evidence for subsurface oceans in Europa and Callisto. *Nature* **395**, 777–780 (1998).
2. M. G. Kivelson, K. K. Khurana, C. T. Russell, M. Volwerk, R. J. Walker, C. Zimmer, Galileo magnetometer measurements: A stronger case for a subsurface ocean at europa. *Science* **289**, 1340–1343 (2000).
3. G. V. Hoppa, B. R. Tufts, R. Greenberg, P. E. Geissler, Formation of cycloidal features on europa. *Science* **285**, 1899–1902 (1999).
4. R. Greenberg, P. Geissler, B. R. Tufts, G. V. Hoppa, Habitability of Europa's crust: The role of tidal-tectonic processes. *J. Geophys. Res. Planets* **105**, 17551–17562 (2000).
5. D. P. O'Brien, P. Geissler, R. Greenberg, A melt-through model for chaos formation on Europa. *Icarus*. **156**, 152–161 (2002).
6. R. T. Pappalardo, M. J. S. Belton, H. H. Breneman, M. H. Carr, C. R. Chapman, G. C. Collins, T. Denk, S. Fagents, P. E. Geissler, B. Giese, R. Greeley, R. Greenberg, J. W. Head, P. Helfenstein, G. Hoppa, S. D. Kadel, K. P. Klaasen, J. E. Klemaszewski, K. Magee, A. S. McEwen, J. M. Moore, W. B. Moore, G. Neukum, C. B. Phillips, L. M. Prockter, G. Schubert, D. A. Senske, R. J. Sullivan, B. R. Tufts, E. P. Turtle, R. Wagner, K. K. Williams, Does Europa have a subsurface ocean? Evaluation of the geological evidence. *J. Geophys. Res. Planets* **104**, 24015–24055 (1999).
7. L. M. Prockter, R. T. Pappalardo, Folds on Europa: Implications for crustal cycling and accommodation of extension. *Science* **289**, 941–943 (2000).
8. F. Nimmo, M. Manga, Geodynamics of Europa's icy shell, in *Europa* (Univ. of Arizona Press, 2017), pp. 381–404.
9. H. Hussmann, T. Spohn, Thermal-orbital evolution of Io and Europa. *Icarus*. **171**, 391–410 (2004).
10. S. A. Kattenhorn, L. M. Prockter, Evidence for subduction in the ice shell of Europa. *Nature Geosci.* **7**, 762–767 (2014).
11. M. A. Hesse, J. S. Jordan, S. D. Vance, A. V. Oza, Does Europa have a subsurface ocean? Evaluation of the geological evidence. *Geophys. Res. Lett.* **49**, 2021GL095416 (2022).
12. C. F. Chyba, C. B. Phillips, Europa as an abode of life. *Orig. Life Evol. Biosph.* **32**, 47–67 (2002).
13. E. P. Turtle, E. Pierazzo, Thickness of a Europan ice shell from impact crater simulations. *Science* **294**, 1326–1328 (2001).

14. P. M. Schenk, Thickness constraints on the icy shells of the galilean satellites from a comparison of crater shapes. *Nature* **417**, 419–421 (2002).
15. V. J. Bray, G. S. Collins, J. V. Morgan, H. J. Melosh, P. M. Schenk, Hydrocode simulation of Ganymede and Europa cratering trends—How thick is Europa's crust? *Icarus*. **231**, 394–406 (2014).
16. R. Cox, A. W. Bauer, Impact breaching of Europa's ice: Constraints from numerical modeling. *J. Geophys. Res. Planets* **120**, 1708–1719 (2015).
17. E. A. Silber, B. C. Johnson, Impact crater morphology and the structure of Europa's ice shell. *J. Geophys. Res. Planets* **122**, 2685–2701 (2017).
18. S. D. Kadel, F. C. Chuang, R. Greeley, J. M. Moore, Geological history of the Tyre region of Europa: A regional perspective on Europan surface features and ice thickness. *J. Geophys. Res. Planets* **105**, 22657–22669 (2000).
19. J. M. Moore, E. Asphaug, M. J. S. Belton, B. Bierhaus, H. H. Breneman, S. M. Brooks, C. R. Chapman, F. C. Chuang, G. C. Collins, B. Giese, R. Greeley, J. W. Head, S. Kadel, K. P. Klaasen, J. E. Klemaszewski, K. P. Magee, J. Moreau, D. Morrison, G. Neukum, R. T. Pappalardo, C. B. Phillips, P. M. Schenk, D. A. Senske, R. J. Sullivan, E. P. Turtle, K. K. Williams, Impact features on Europa: Results of the Galileo Europa Mission (GEM). *Icarus*. **151**, 93–111 (2001).
20. J. M. Moore, E. Asphaug, R. J. Sullivan, J. E. Klemaszewski, K. C. Bender, R. Greeley, P. E. Geissler, A. S. McEwen, E. P. Turtle, C. B. Phillips, B. R. Tufts, J. W. Head, R. T. Pappalardo, K. B. Jones, C. R. Chapman, M. J. S. Belton, R. L. Kirk, D. Morrison, Large impact features on Europa: Results of the Galileo nominal mission. *Icarus.* **135**, 127–145 (1998).
21. K. N. Singer, W. B. McKinnon, P. M. Schenk, Ice lithosphere thickness on Europa from impact basin ring-graben, in *44th Lunar and Planetary Science Conference (LPSC),* abstract 2197 (2013).
22. K. N. Singer, W. B. McKinnon, P. M. Schenk, Thin ice lithospheres and high heat flows on Europa from large impact structure ring-graben. *J. Geophys. Res. Planets.* **218**, e2023JE007928 (2023).
23. H. J. Melosh, *Impact Cratering: A Geologic Process* (Oxford Univ. Press, 1989).
24. H. J. Melosh, W. B. McKinnon, The mechanics of ringed basin formation. *Geophys. Res. Lett.* **5**, 985–988 (1978).
25. W. B. McKinnon, H. J. Melosh, Evolution of planetary lithospheres: Evidence from multiringed structures on ganymede and callisto. *Icarus*. **44**, 454–471 (1980).

26. B. C. Johnson, D. M. Blair, G. S. Collins, H. J. Melosh, A. M. Freed, G. J. Taylor, J. W. Head, M. A. Wieczorek, J. C. Andrews-Hanna, F. Nimmo, J. T. Keane, K. Miljković, J. M. Soderblom, M. T. Zuber, Formation of the Orientale lunar multiring basin. *Science* **354**, 441–444 (2016).
27. E. Bjonnes, B. C. Johnson, A. J. Evans, Estimating Venusian thermal conditions using multiring basin morphology. *Nat. Astron*. **5**, 498–502 (2021).
28. B. C. Johnson, J. C. Andrews-Hanna, G. S. Collins, A. M. Freed, H. J. Melosh, M. T. Zuber, Controls on the formation of lunar multiring basins. *J. Geophys. Res. Planets* **123**, 3035–3050 (2018).
29. A. A. Amsden, H. M. Ruppel, C. W. Hirt, SALE: A simplified ALE computer program for fluid flow at all speeds. *Los Alamos National Laboratories Report.* LA-8095 (1980).
30. G. S. Collins, H. J. Melosh, B. A. Ivanov, Modeling damage and deformation in impact simulations. *Meteorit. Planet. Sci.* **39**, 217–231 (2004).
31. K. Wünnemann, G. S. Collins, H. J. Melosh, A strain-based porosity model for use in hydrocode simulations of impacts and implications for transient crater growth in porous targets. *Icarus.* **180**, 514–527 (2006).
32. L. G. J. Montési, M. T. Zuber, A unified description of localization for application to large-scale tectonics. *J. Geophys. Res. Planets* **107**, 2001JB000465 (2002).
33. G. S. Collins, Numerical simulations of impact crater formation with dilatancy. *J. Geophys. Res. Planets* **119**, 2600–2619 (2014).
34. K. Zahnle, P. Schenk, H. Levison, L. Dones, Cratering rates in the outer Solar System. *Icarus*. **163**, 263–289 (2003).
35. G. S. Collins, H. J. Melosh, R. A. Marcus, Earth impact effects program: A web-based computer program for calculating the regional environmental consequences of a meteoroid impact on Earth. *Meteorit. Planet. Sci.* **40**, 817–840 (2005).
36. H. C. F. C. Hay, G. S. Collins, T. M. Davison, Complex crater collapse: A comparison of the block and Melosh models of acoustic fluidization, in *45th Lunar and Planetary Science Conference (LPSC)*, abstract #1938 (2014).
37. L. E. Senft, S. T. Stewart, Dynamic fault weakening and the formation of large impact craters. *Earth Planet. Sci. Lett.* **287**, 471–482 (2009).
38. E. Carnahan, S. D. Vance, R. Cox, M. A. Hesse, Surface-to-ocean exchange by the sinking of impact generated melt chambers on Europa. *Geophys. Res. Lett.* **49**, e2022GL100287 (2023).

39. H. J. Melosh, A simple mechanical model of Valhalla basin, Callisto, *J. Geophys. Res. Solid Earth* **87**, 1880–1890 (1982).
40. P. M. Schenk, The geology of Callisto. *Geophys. Res. Planets.* **100**, 19023–19040 (1995).
41. R. Greeley, P. H. Figueredo, D. A. Williams, F. C. Chuang, J. E. Klemaszewski, S. D. Kadel, L. M. Prockter, R. T. Pappalardo, J. W. Head III, G. C. Collins, N. A. Spaun, R. J. Sullivan, J. M. Moore, D. A. Senske, B. R. Tufts, T. V. Johnson, M. J. S. Belton, K. L. Tanaka, Geologic mapping of Europa. *J. Geophys. Res. Planets* **105**, 22559–22578 (2000).
42. F. Nimmo, P. C. Thomas, R. T. Pappalardo, W. B. Moore, The global shape of Europa: Constraints on lateral shell thickness variations. *Icarus.* **191**, 183–192 (2007).
43. E. B. Bierhaus, K. Zahnle, C. R. Chapman, R. Dotson, Europa's crater distributions and surface ages, in *Europa* (Univ. of Arizona Press, 2009), pp. 161–180.
44. W. B. McKinnon, Convective instability in Europa's floating ice shell. *Geophys. Res. Lett.* **26**, 951–954 (1999).
45. G. S. Collins, D. Elbeshausen, T. M. Davison, K. Wünnemann, B. Ivanov, H. J. Melosh, iSALE-Dellen manual (2016).
46. B. A. Ivanov, D. Deniem, G. Neukum, Implementation of dynamic strength models into 2D hydrocodes: Applications for atmospheric breakup and impact cratering. *Int. J. Impact Eng.* **20**, 411–430 (1997).
47. H. J. Melosh, E. V. Ryan, E. Asphaug, Dynamic fragmentation in impacts—Hydrocode simulation of laboratory impacts. *J. Geophys. Res. Planets* **97**, 14 (1992).
48. J. H. Tillotson, Metallic equations of state for hypervelocity impact (General Atomic Report GA-3216. Technical Report, 1962).
49. B. A. Ivanov, F. Langenhorst, A. Deutsch, U. Hornemann, How strong was impact-induced CO_2 degassing in the Cretaceous-Tertiary event? Numerical modeling of shock recovery experiments, in *Catastrophic Events and Mass Extinctions: Impacts and Beyond*, C. Koeberl, K. G. MacLeod, Eds. (Geological Society of America, 2002), vol. 356, pp. 587–594.
50. S. Lowry, A. Fitzsimmons, P. Lamy, P. Weissman, Kuiper belt objects in the planetary region: The Jupiter-family comets, in *The Solar System Beyond Neptune* (Univ. of Arizona Press, 2008), pp. 397–410.
51. C. Snodgrass, A. Fitzsimmons, S. C. Lowry, P. Weissman, The size distribution of Jupiter Family comet nuclei. *MNRAS* **414**, 458–469 (2011).

52. V. J. Bray, "Impact crater formation on the icy Galilean satellites," thesis, Imperial College London, London (2009).
53. M. Beeman, W. B. Durham, S. H. Kirby, Friction of ice. *J. Geophys. Res.* **93**, 7625–7633 (1988).
54. W. B. Durham, H. C. Heard, S. H. Kirby, Experimental deformation of polycrystalline H_2O ice at high pressure and low temperature: Preliminary results. *J. Geophys. Res.* **88**, B377 (1983).
55. D. Elbeshausen, H. J. Melosh, A nonlinear and time-dependent visco-elasto-plastic rheology model for studying shock-physics phenomena. *Eng. Rep.* **2**, e12322 (2020).
56. N. Yasufuku, S. M. Springman, L. U. Arenson, T. Ramholt, Stress-dilatancy behaviour of frozen sand in direct shear, in *Permafrost* (Balkema, 2003), pp. 1253–1258.
57. Bland, M. T., McKinnon, W. B. Forming Ganymede's grooves at smaller strain: Toward a self-consistent local and global strain history for Ganymede. *Icarus.* **245**, 247–262 (2015).
58. Melosh, H. J. Acoustic fluidization: A new geologic process? *J. Geophys. Res. Solid Earth* **84**, 7513–7520 (1979).
